# Engaging with stakeholders for community-based health research in India: Lessons learnt, challenges and opportunities

**DOI:** 10.7189/jogh.11.03072

**Published:** 2021-05-01

**Authors:** Rutuja Patil, Dhiraj Agarwal, Harshpreet Kaur, Mukta Gadgil, Tracy Jackson, Genevie Fernandes, Sanjay Juvekar

**Affiliations:** 1 Vadu Rural Health Program, KEM Hospital Research Centre Pune, Pune, India; 2 NIHR Global Health Research Unit on Respiratory Health (RESPIRE), Usher Institute of Population Health Science and Informatics, University of Edinburgh, Edinburgh, UK; 3 State Health Systems Resource Centre (SHSRC), Pune, India; 4 State Family Welfare Bureau, Pune, India

Stakeholders are individuals, organisations or communities who are responsible for, or affected by, the processes or outcome of the research [1]. Stakeholder engagement (SE) in health research has the potential to inform quality improvements by incorporating multiple perspectives of the stakeholders beyond the traditional research team [2]. Increasingly, funders are acknowledging the benefits of SE on research outcomes and mandating it on grant applications [2]. It can help to improve the health, knowledge and well-being of communities by decreasing the ambiguity surrounding research findings and increasing early acceptance of the research findings [3]. Early SE could help obtain funding and facilitate in reducing the gap between research to policy by creating research that is of benefit and interest to numerous stakeholders [4,5].

Current descriptions and evaluations of SE, highlight the need for establishing more SE process methods [6]. Redundancies, lack of knowledge to establish priorities based on stakeholders needs, inadequate reporting of study-results and ambiguous study designs, result in the wastage of 85% of investment in health and biomedical research every year [7,8]. Although financial and theoretical support for research demands SE, its impact has not been well-recognised or established [2]. Moreover, the terminologies, guidelines and concepts for reporting SE process and outcomes in the health research provide little published evidence on the best SE practices [2].

Despite the positive potential impacts of SE, limited information is available on SE processes especially within a South Asian context [4,9]. The concept of SE is well recognised in high-income countries and researchers from low-middle-income-countries (LMICs) are slowly acknowledging the importance of conducting meaningful SE [1,4]. In this article we discuss the SE process employed at the Vadu Rural Health Program (VRHP), a department of KEM Hospital Research Centre Pune (KEMHRC), when conducting community-based public health research in rural areas, and describe our experiences and challenges in conducting SE activities.

## STAKEHOLDER ENGAGEMENT AT VRHP

In countries like India, where the state governments govern health and central government develop policies, the policy dialogue must happen at National, State and Community levels. For effective implementation and wider reach, VRHP seeks to engage stakeholders at these three levels including: study participants, patients, communities, government health authorities, researchers and funders. Engaging stakeholders in research prioritisation enables us to design and produce research that is beneficial and relevant to those impacted by its outcomes. This increases utilisation of research findings and reduces the time in implementing policy recommendations into practice as the research is based on the stakeholders’ needs.

**Figure:**
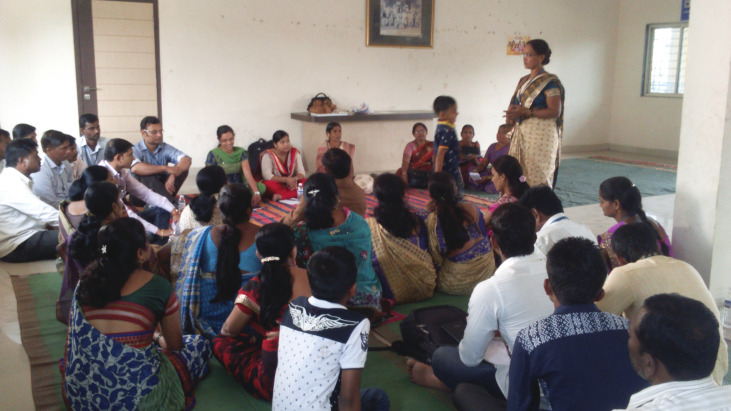
Photo: Field Research Assistant conducting community meeting in study area (source: VRHP, KEMHRC collection, used with permission).

VRHP has been conducting community-based research for the last five decades, yet SE was only recently planned systematically. Historically, VHRP has worked closely with local communities in areas where it conducts its public health research studies namely, administrative blocks of Ambegaon, Haveli, Junnar, Khed and Shirur in Pune district. These cover more than 600 villages and nearly a million people. SE activities were undertaken to fulfil VRHP’s mission “*to provide evidence-based, sustainable and rational health care solutions for the rural population using globally relevant community-based ethical research*”.

## IDENTIFYING STAKEHOLDERS

We identified the stakeholders at three tiers: community; health provider & researcher and policymakers. The community tier includes study participants, patients and their families, local leadership and populations residing in the study area. The second tier incorporates public and private health care providers, program implementers and the research fraternity. The third tier involves the policy (decision) makers, media, national and international health organisations, and funding agencies. These stakeholders are distinguished based on the power and impact matrix described in **Table 1**.

**Table 1:** Stakeholders matrix with their impact and policy

	Will have low impact and low stake in the research	Will have a high impact and high stake in the research
**High power to block or create change**	Grampanchayat representative (Local Government), Indian Medical Association, Government agencies (ICMR, CSIR, DBT, DST)	Study participants, community members, Public health systems (District and state), Funders, Health care providers in study area (PHC, RH, Private clinics), Ethics Committees and regulatory bodies
**Low power to block or create change**	Researcher (Study team member), Clinician (Study team member), Researchers (non-study team members)	Local newspaper/news channel representative (Reporter), Community platforms (self-help groups, youth groups), Social media, Advisory bodies like Scientific Advisory Committee

## CONDUCTING STAKEHOLDER ENGAGEMENT

VHRP used three strategies to engage with the first tier of stakeholders: community. Initially, local communities were regularly engaged using traditional methods such as interpersonal communication (community meetings, banners, posters) and media events (screening and discussion of educational films including documentaries, street plays, rallies, and cross-country events). Over time, VHRP refined its unique model of community involvement, whereby local community members were invited and engaged as partners throughout the research process. We first identified interested and dynamic locals from the community, provided them with voluntary or paid roles within VHRP and trained them to undertake research in their own communities. We chose these representatives based on their interest and rapport with their local community, not solely on their qualifications. Through our flagship Health and Demographic Surveillance System, we have been actively collecting information on communities’ health statuses that informs us about communities’ needs.

After training, community representatives worked closely with the VHRP research team in the conceptualisation and design of research studies, and further contributed to planning and implementation. Involving community representatives in analysis and dissemination provided community-specific context to the research process. To enhance institutional capacity in SE and develop systematic strategies, VRHP recently established a Community Engagement Committee (CEC) that comprises Scientists, Programme Managers, Field Supervisors and Field Volunteers. The CEC is responsible for planning, conducting, and evaluating SE activities. It regularly provides information and solicits feedback on completed, ongoing and proposed research studies to influential community members like the local village-level elected leaders, namely the ‘Sarpanch’ and ‘Deputy Sarpanch’ and members of the Gram Panchayat (local self-government) along with public community health workers called ASHAs (Accredited Social Health Activists), Auxiliary Nurse Midwives (ANM), Gram Sevak (village development officer from the government) and villagers [10].

At the second tier, we involved a wide range of health providers, including public and private practitioners at the primary and secondary health care level and researchers. Engagement with each group was contextualised depending on their role and interest. We conducted regular Continuous Medical Education (CME) for all medical practitioners in the study area and provided training sessions for the implementers, like ASHAs and ANMs, based on the study needs. This bottom-up approach has streamlined interactions with implementers; percolating knowledge and processes to the public health department staff, thus creating meaningful collaboration with the health care providers. These activities have provided stakeholders with ownership of the research process, increasing their commitment to the success of the research. Further, the regulatory authorities like ethics committees, scientific community, including the advisors and other researchers, form an integral section of stakeholders of this tier. The stakeholders are engaged using the most common modes of communication like scientific reports, publications and presentations.

At the third tier, we have been directly working with the government, which comprises the significant chunk of tier-three, to effectively implement many relevant local and national public health programs. This tier includes district and state-level managers of public health programmes and senior government bureaucratic officials in state and national level public health departments. The top-down approach has facilitated these engagements. Communication with policy and decision-makers is two-way facilitating mutual learnings and research transparency. Engagement with this tier has played an important role in devising the research priorities and strategies, especially for implementation and operational research.

**Figure 1:**
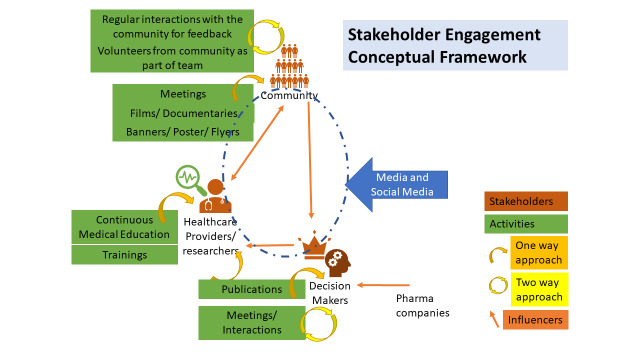
Conceptual framework for stakeholder engagement.

Further, utilising media and social media has provided access to a broader audience of previously unreached stakeholders. Research publications and conference presentations enhance credibility of the research and engage the research communities, including collaborators and funding agencies. Additionally, pharma companies play an important role in influencing the third tier and thus affect all aspects of stakeholder engagement. However, as pharma companies have minimal influence on the research we conduct, we did not consider them our direct stakeholders (**Figure 1**).

## CHALLENGES

We faced a number of challenges, the primary being lack of awareness. Various stakeholders were unaware how their involvement would improve the research, and community members were unclear how engagement activities would benefit them, so initially were reluctant to get involved. Researchers were also unaware of the importance of including meaningful SE in research projects and there were a lack of effective SE policy/action plans available. Stakeholder engagement is often underfunded, our NIHR Global Health Research Unit on Respiratory Health, (RESPIRE) funding was the first SE-specific funding we have received since 2018.

## PRACTICAL CONSIDERATIONS

Regular communication with stakeholders, not only when dictated by the research project, and transparency on the outcomes of their support helped maintain meaningful engagement with stakeholders. Respect towards them, their time and a collaborative approach created a mutual appreciation between researchers and stakeholders. Additionally, supplying culturally appreciated foods and arranging stakeholder meetings at convenient times and places were essential for successful SE activities. Providing incentives for engagement, or compensation for travel or loss of wages determined certain stakeholders’ participation in SE activities. SE-allocated funding from RESPIRE ensured dedicated SE personnel and resources which increased the feasibility of conducting SE activities. Having a SE committee or CEC at each research institute added value. Capacity-building among researchers by training or giving other opportunities or exposure would help to generate SE champions. Further, a national-level committee of policymakers and other appropriate contacts of the potential stakeholders would be useful for SE activities in India.

## CONCLUSIONS

These experiences from VRHP KEMHRC can be used by any other organisations working in similar activities of community-based research.
